# Multi-Label Feature Selection with Conditional Mutual Information

**DOI:** 10.1155/2022/9243893

**Published:** 2022-10-08

**Authors:** Xiujuan Wang, Yuchen Zhou

**Affiliations:** ^1^Faculty of Information and Technology, Beijing University of Technology, Beijing 100020, China; ^2^Beijing-Dublin International College, Beijing University of Technology, Beijing 100020, China

## Abstract

Feature selection is an important way to optimize the efficiency and accuracy of classifiers. However, traditional feature selection methods cannot work with many kinds of data in the real world, such as multi-label data. To overcome this challenge, multi-label feature selection is developed. Multi-label feature selection plays an irreplaceable role in pattern recognition and data mining. This process can improve the efficiency and accuracy of multi-label classification. However, traditional multi-label feature selection based on mutual information does not fully consider the effect of redundancy among labels. The deficiency may lead to repeated computing of mutual information and leave room to enhance the accuracy of multi-label feature selection. To deal with this challenge, this paper proposed a multi-label feature selection based on conditional mutual information among labels (CRMIL). Firstly, we analyze how to reduce the redundancy among features based on existing papers. Secondly, we propose a new approach to diminish the redundancy among labels. This method takes label sets as conditions to calculate the relevance between features and labels. This approach can weaken the impact of the redundancy among labels on feature selection results. Finally, we analyze this algorithm and balance the effects of relevance and redundancy on the evaluation function. For testing CRMIL, we compare it with the other eight multi-label feature selection algorithms on ten datasets and use four evaluation criteria to examine the results. Experimental results illustrate that CRMIL performs better than other existing algorithms.

## 1. Introduction

In the era of big data, data in all fields are increasing explosively [1–3]. Therefore, feature selection has rapidly become a hot topic. Proper feature selection can improve the efficiency and accuracy of classifiers. Compared with the traditional single-label feature selection, multi-label feature selection is more suitable for solving problems in the real world [4]. Therefore, multi-label feature selection applies to various fields, such as image processing [5, 6], text categorization [7, 8], and bioinformatics [9].

Multi-label feature selection algorithms usually consider how to reduce the influence of redundancy among information. The commonly used processing methods include the swarm intelligence algorithm [10], which regards features as individuals and a group of features as populations for reproduction, evolution, and mutation to reduce the redundancy of information and improve the algorithm's accuracy. Another idea is manifold learning [11]. This approach can diminish useless features for classifiers from the perspective of dimension reduction. Considering the relevance between features and labels by calculating mutual information between features and labels is another approach [12]. This method can help judge which features need to be kept. Much prior work has proved that mutual information is an efficient method to extract features [13, 14]. Because mutual information is more concise and effective [15], this paper will explore multi-label feature selection based on mutual information.

Many multi-label feature selection algorithms have been based on mutual information [16–18]. Once the mutual information of two different features or two labels is greater than zero, redundancy appears. Although these algorithms have considered the relevance between features and labels, and the redundancy among features, they do not adequately process the redundancy among labels, eventually leading to an unsatisfactory result. This paper proposes a new approach to deal with the redundancy among labels and a multi-label feature selection based on this approach.

The rest of the paper reads as follows: In Section 2, the related work is summarized. We then propose a new multi-label feature selection algorithm in Section 3. In Section 4, relevant experiments prove the efficiency of the proposed algorithm. In Section 5, we summarize this paper and explain the directions of future work.

In summary, the study offers the following contributions:

- We propose a new method to avoid repeating calculations on redundant label information.
- We propose a novel algorithm of multi-label feature selection and get good results. It performs better on most datasets, which have redundancy among labels.
- We set many experiments from different perspectives to test the proposed algorithms; some of them are innovative.

## 2. Related Work

In the early stage of multi-label feature selection, most proposed algorithms transform multi-label datasets into multiple single-label datasets and process all single-label datasets with traditional single-label feature selection algorithms. For example, literature [19] divides a dataset *D* into *q* independent 01 datasets by Binary Relevance (BR) and transforms each possible label combination into unique classes by Label Powerset (LP). Then this paper deals with new datasets by Relief and traditional single-label feature selection algorithm based on mutual information. However, this kind of algorithm cannot work on large datasets. To overcome this challenge, the literature [20] pruned the labels that infrequently appeared in datasets. This approach can reduce the size of final datasets. However, this algorithm only transforms multi-label datasets into many single-label datasets. which may ignore the effects between features and features, labels and labels in the original datasets.

In recent years, many algorithm adaptation methods have been applied to high-dimension feature selection. For example, the literature [21] details two stages to implement feature selection of gene datasets. A greedy approach is used to assign the maximum number of samples to different gene classes in the first step. In the second step, clustering and lasso methods are selected to extract the remaining features. Additionally, Deep Neural Network is embedded into a high-dimension feature selection method [22]. To reduce the effects of outliers and noise in datasets, the literature [23] proposes Unsupervised Feature Selection with Robust Data Reconstruction (UFS-RDR) by minimizing the graph regularized weighted data reconstruction error function. The relevant estimation tools are also developed. To evaluate the stability of high-dimension feature selection approaches, the literature [24] proposes a novel estimator considering inter-intrastability of subsets. These high-dimension feature selection algorithms provide ideas for multi-label feature selection. Particularly, multi-label feature selection based on mutual information attracts extensive attention. The literature [25] has considered the interaction between selected features and unselected features and proposed MDMR as follows:

(1) $$\begin{array}{c} J\left(f_{k}\right)=\underset{f_{i}\in S}{\sum }\underset{l_{i}\in L}{\sum }\left[I\left(f_{k},l_{i}\right)-I\left(f_{k},l_{i},f_{j}\right)\right], \end{array}$$

where *S* is the selected feature set and *L* is the label set. The literature [26] considers redundancy when computing the relevance between features and labels. This paper regards redundancy existing among information as part of the relevance, which means that

(2) $$\begin{array}{c} \text{Redundancy}=\mathrm{Relevance*}C. \end{array}$$

The coefficient *C* should become greater when the selected features are strongly dependent on other features, and conversely, *C* should become smaller. Therefore, *I*(*f*_*k*_, *f*_*i*_) can be a part of *C*. Additionally, because *C* ∈ (0, 1), *H*(*f*_*k*_) is used to normalize *I*(*f*_*k*_, *f*_*i*_). As a result, the selected feature can be described as

(3) $$\begin{array}{c} \underset{f_{k}\in \left\{F-S_{i-1}\right\}}{\text{max}}\left[\underset{l_{i}\in L}{\sum }I\left(f_{k},l_{i}\right)-\underset{f_{i}\in S_{i-1}}{\sum }\frac{I\left(f_{k},f_{i}\right)}{H\left(f_{k}\right)}*\underset{l_{i}\in L}{\sum }I\left(f_{k},l_{i}\right)\right]. \end{array}$$

However, the algorithm directly computes the relevance and redundancy without further processing. This method might lead to the effects of relevance and redundancy being unbalanced. To solve this problem, the literature [27] proposes granular feature selection, which transforms features into granular feature groups. After computing the relevance and redundancy, the results divide by the size of related sets. This idea can be detailed by the following formula:

(4) $$\begin{array}{c} J\left(f_{k}\right)=\frac{1}{\left|G\right|}\underset{l_{i}\in G}{\sum }I\left(f_{k},l_{i}\right)-\frac{1}{\left|S\right|}\underset{f_{i}\in S}{\sum }I\left(f_{k},f_{i}\right), \end{array}$$

where |*G*| is the granularity. However, these algorithms do not consider the redundancy among labels. Literature [15, 28] achieves better results after considering the redundancy among labels. The algorithm can be described as formula (5), respectively.

(5) $$\begin{array}{c} J\left(f_{k}\right)=\underset{l_{i}\in L}{\sum }\left\{\underset{l_{i}\neq l_{j},l_{j}\in L}{\sum }I\left(f_{k},l_{j}|l_{i}\right)-\frac{1}{\left|S\right|}\underset{f_{j}\in S}{\sum }I\left(f_{k};f_{j}\right)\right\}. \end{array}$$

Although the redundancy among labels has been considered, the redundant information may be accumulated more than once. This problem is detailed in Section 3, and we propose a solution in that section.

## 3. Multi-Label Feature Selection considering Redundancy on Mutual Information of Labels (CRMIL)

Firstly, a problem in traditional multi-label feature selection is introduced. Many multi-label feature selection algorithms, which are proposed for solving this problem, have shortages. To improve the accuracy, we propose a new method to compute the redundancy among labels. This method can reduce the redundancy among labels and calculate the relevance between features and labels. Then, the redundancy among features is computed. Finally, we propose the new multi-label feature selection algorithm and detail the pseudocode.

### 3.1. A Problem

**Figure 1:**
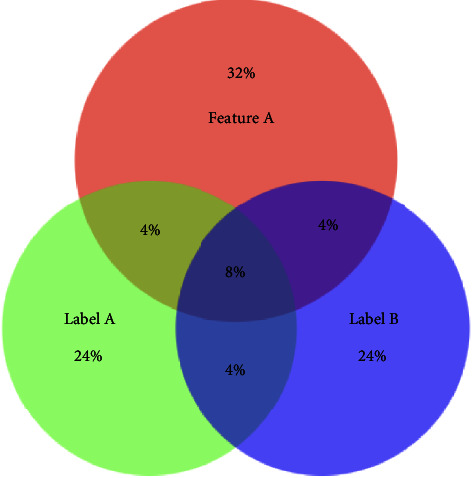
Venn diagram detailing Feature A Label A and Label B.

**Figure 2:**
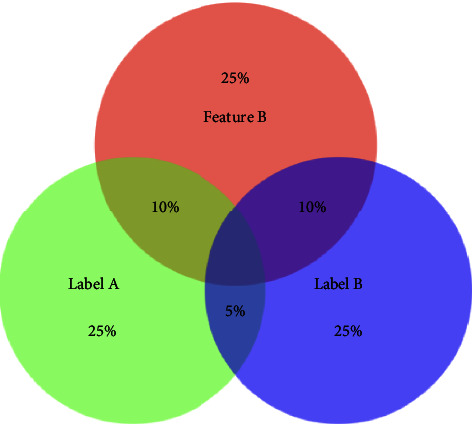
Venn diagram detailing Feature B Label A and Label B.

Traditional multi-label feature selection, which does not consider redundancy among labels, might encounter the following problem:

In Figure 1 and 2 show that Feature A and Feature B contain 16% and 20% of useful information, respectively. Feature B should be selected. If the redundancy among labels is ignored, the valuable information provided by Feature A and Feature B is 24% and 20%, respectively. As a result, Feature A will be selected due to the redundancy among labels. After considering the redundancy among labels, the mutual information between features and labels is 16% and 20%, respectively. Feature B will be selected. Therefore, the redundancy among labels is worth considering. The following parts will focus on how we design the multi-label feature selection algorithm considering the redundancy.

### 3.2. Multi-Label Conditional Mutual Information

Existing multi-label feature selection algorithms usually use conditional mutual information to calculate the redundancy among labels. In the literature [15, 28], *I*(*f*, *l*_*i*_*|l*_*j*_) is essential to compute the redundancy among labels. However, these algorithms enumerate every label as a condition and sum up all conditional mutual information. The sum can be regarded as the relevance between features and labels with diminishing redundancy among labels, such as formula (6). Once more than two labels contain the same information, the overlapping information will be counted more than once. This situation may reduce the accuracy of the result.

(6) $$\begin{array}{c} \underset{l_{i}\in L}{\sum }\underset{l_{j}\in L,l_{j}\neq l_{i}}{\sum }I\left(f,l_{i}|l_{j}\right), \end{array}$$

where *f* is the pending feature, *l*_*i*_ and *l*_*j*_ are the label elements that are different at any time. Formula (6) has been proved and detailed in the literature [22].

We propose that regarding part of the label set as conditions on mutual information can overcome this challenge. In the proposed multi-label feature selection algorithm, the relevant part which computes the redundancy among labels, can be detailed in the following formulafd7:

(7) $$\begin{array}{c} \underset{l_{i}\in L}{\sum }I\left(f,l_{i}|Y\right), \end{array}$$

where *Y*={*l*_*j*_*|l*_*j*_ ∈ *L*,  *l*_*j*_ ≠ *l*_*i*_}. This can reduce the effects of the redundancy among labels.

**Proof**

(8) $$\begin{array}{c} \underset{l_{i}\in L}{\sum }I\left(f,l_{i}|Y\right)=\underset{l_{i}\in L}{\sum }H\left(l_{i}|Y\right)-H\left(l_{i}|f,Y\right)\\ \left.\;=\underset{l_{i}\in L}{\sum }\underset{y\in L}{\sum }\underset{x\in l_{i}}{\sum }\left(p\left(x,y\right)*logp\left(x|y\right)\right.+\underset{z\in f}{\sum }p\left(x,y,z\right)*logp\left(x|y,z\right)\right) \end{array}$$

This shows that, compared to the traditional formula (6), formula (7) does not sum every element of every label in label sets. Therefore, this method calculates the better result in Section 3.1. Formula (7) thus can avoid the repeated calculation on information that many labels contain.

### 3.3. Alleviate the Redundancy among Features

After considering the redundancy among labels, the proposed algorithm calculates the redundancy among features. Mutual information can reflect the total information shared by two random variables. In feature selection, features can be seen as random variables. Therefore, we regard the mutual information of all pairs of features as the redundancy among features. Then, when a new feature is selected, the redundancy of features is computed by the following formulafd9:

(9) $$\begin{array}{c} \text{Red}\left(f,S\right)=\underset{f_{i}\in S}{\sum }I\left(f,f_{i}\right), \end{array}$$

where *f* is a pending feature.

### 3.4. Proposed Algorithms

Based on above proofs, features with larger value on formula (8) and less value on formula (9) should be selected. After analyzing the relevance and redundancy of information, we use the size of the label (*α*/|*L*|) and the selected feature set (*β*/|*S*|) to balance the effect of relevance and redundancy on the results. *α* and *β* are used to affect the importance of the label set and the selected feature set, respectively. We choose *α*=*β*=1 (this will be proved in Section 4.3). Finally, we proposed a new multi-label feature selection algorithm (CRMIL). The evaluation function can be defined as follows:

(10) $$\begin{array}{c} \begin{array}{c} J\left(f_{k}\right)=\frac{1}{\left|L\right|}\underset{l_{i}\in L}{\sum }I\left(f_{k},l_{i}|Y\right)-\frac{1}{\left|S\right|}Red\left(f_{k},S\right)\\ =\frac{1}{\left|L\right|}\underset{l_{i}\in L}{\sum }I\left(f_{k},l_{i}|Y\right)-\frac{1}{\left|S\right|}\underset{f_{i}\in S}{\sum }I\left(f_{k},f_{i}\right)\; \end{array}\; \end{array}$$

where *Y*={*l*_*j*_*|l*_*j*_ ∈ *L*,  *l*_*j*_ ≠ *l*_*i*_} and *f*_*k*_ is a pending feature.

**Property 1 .**

*J*(*f*_*k*_) ∈ (−1, 1).

**Proof**

(11) $$\begin{array}{c} \;\begin{array}{c} ∵I\left(f_{k},l_{i}|Y\right)\in \left(0,1\right),I\left(f_{k},f_{i}\right)\in \left(0,1\right)\\ ∴\frac{1}{\left|L\right|}\underset{l_{i}\in L}{\sum }I\left(f_{k},l_{i}|Y\right)\in \left(0,1\right),\frac{1}{\left|S\right|}\underset{f_{i}\in S}{\sum }I\left(f_{k},f_{i}\right)\in \left(0,1\right)\\ ∴J\left(f_{k}\right)=\frac{1}{\left|L\right|}\underset{l_{i}\in L}{\sum }I\left(f_{k},l_{i}|Y\right)-\frac{1}{\left|S\right|}\underset{f_{i}\in S}{\sum }I\left(f_{k},f_{i}\right)\in \left(-1,1\right)\; \end{array}\; \end{array}$$

**Property 2 .**

*J*(*f*_*k*_) ∈ (−1, 0), when most of the relevance between features and labels satisfies 0 < *I*(*f*_*k*_, *l*_*i*_*|Y*) < *α* (*α*⟶0) and most of the redundancy among features satisfies 1 − *α* < *I*(*f*_*k*_, *f*_*i*_) < 1.

**Proof**

(12) $$\begin{array}{c} \begin{array}{c} ∵I\left(f_{k},l_{i}|Y\right)\in \left(0,\alpha \right),I\left(f_{k},f_{i}\right)\in \left(1-\alpha ,1\right)\\ ∴\frac{1}{\left|L\right|}\underset{l_{i}\in L}{\sum }I\left(f_{k},l_{i}|Y\right)\in \left(0,\alpha \right),\frac{1}{\left|S\right|}\underset{f_{i}\in S}{\sum }I\left(f_{k},f_{i}\right)\in \left(1-\alpha ,1\right)\\ ∴J\left(f_{k}\right)=\frac{1}{\left|L\right|}\underset{l_{i}\in L}{\sum }I\left(f_{k},l_{i}|Y\right)-\frac{1}{\left|S\right|}\underset{f_{i}\in S}{\sum }I\left(f_{k},f_{i}\right)<0\; \end{array}\; \end{array}$$

However, this is hardly the case in normal datasets.

**Property 3 .**

Because the size of datasets is considered, in normal datasets, *J*(*f*_*k*_) ∈ (0, 1).

**Proof**

(13) $$\begin{array}{c} ∵\frac{1}{\left|S\right|}\underset{f_{i}\in S}{\sum }I\left(f_{k},f_{i}\right)<\frac{1}{\left|L\right|}\underset{l_{i}\in L}{\sum }I\left(f_{k},l_{i}|Y\right)<1\\ ∴J\left(f_{k}\right)=\frac{1}{\left|L\right|}\underset{l_{i}\in L}{\sum }I\left(f_{k},l_{i}|Y\right)-\frac{1}{\left|S\right|}\underset{f_{i}\in S}{\sum }I\left(f_{k},f_{i}\right)\in \left(0,1\right) \end{array}$$

In the beginning, *S* is empty. To choose *k* features, we need *k* steps. In every step, we choose the feature with the largest *J*(*f*_*k*_). Then we put the selected feature into *S* and delete the feature from the label set. Finally, the output is a k-dimension vector containing the index of selected features.

### 3.5. Pseudocode

The proposed algorithm requires a feature set *F*, a label set *L*, and the number of features *K* and returns the number set of selected features. Lines 1–2: initializing the number set of selected features and the number of selected features *k*. Lines 3–7: preprocessing the relevance between features and labels in formula (7). Lines 8–22: selecting *k* features by iterating. Among these lines, lines 9–10 select the first feature. The feature with the greatest relevance is selected because there is no element in the selected feature set. Lines 12–17: the redundancy among features is calculated by using formula (8). Lines 18–20: after selecting a feature, the feature needs to be added to the selected feature set and deleted from the original feature set. Finally, the number set of selected features is returned.

### 3.6. Time Complexity Analysis

In the following explanation, *N* is the number of samples, |*F*| is the number of features, and |*L*| is the number of labels. The time complexity of the proposed algorithm is up to three main parts. Firstly, processing the mutual information among features needs to enumerate two different features. This step consumes *O*(|*F*|^2^). Calculating information entropy needs *O*(*N*). Therefore, this part consumes *O*(*N*|*F*|^2^). Secondly, the proposed algorithm preprocesses the relevance between features and labels, which is the main part of the algorithm. Enumerating every feature and label consumes *O*(|*F*‖*L*|), and computing the conditional mutual information consumes *O*(*N*|*L*|). Therefore, the time complexity of this part is *O*(*N*|*L*|^2^|*F*|). Thirdly, the algorithm needs to select *K* features. In every selection, pending features and selected features need to be enumerated simultaneously, which consumes *O*(|*F*|^2^) at most. Therefore, the upper-bound time complexity limit on this part is *O*(*K*|*F*|^2^). As a result, the algorithm's time complexity should be max(*O*(*N*|*F*|^2^), *O*(*N*|*L*|^2^|*F*|)), which depends on the kinds of data in the datasets.

As the time complexity test of a prior work [29], we use Intel(R) Core(TM) i9-9880H CPU @ 2.30 GHz to test the time cost on different datasets. All results are the average level after five times calculations. For example, when a dataset consists of 850 instances, 1000 features, and 50 labels, it takes on average 9.2 s. The number of instances in the dataset then is doubled and the dataset costs around 17.3 s. Furthermore, if the number of features is compressed by half, the time needed is around 2.1 s. These prove that, in reality, the analysis of time complexity is right with great possibility.

## 4. Experimental Results

In this section, we illustrate the adaptability of CRMIL on various datasets and list the experimental results. Firstly, four evaluation criteria are explained. Then we use ten different datasets (Corel5k, Delicious, Flags, Medical, Scene, Enron, GenBase, Social, Yeast, and Emotions) to test CRMIL and compare CRMIL with eight traditional multi-label feature selection algorithms, which are SCLS [26], D2F [30], FIMF [31], PMU [3], AMI [32], NMDG [33], FSSL [34], and MFS-MCDM [35].

### 4.1. Evaluation Criteria

This paper uses four evaluation criteria to examine the results of multi-label feature selection: Hamming Loss, Average Precision, One Error, and Ranking Loss. These criteria are usually used by multi-label feature selection papers [36, 37]. Hamming Loss can be defined as follows:

(14) $$\begin{array}{c} \text{Hamming.Loss}=\frac{1}{\left|D\right|}\overset{\left|D\right|}{\underset{i=1}{\sum }}\frac{L_{i}^{\prime }⊕L_{i}}{\left|L\right|}, \end{array}$$

where *L*_*i*_′ is the predicted label for every sample, *L*_*i*_ is the real label for every sample, and ⊕ is the XOR operation. Hamming Loss reflects the misclassification of every single-label. The lower Hamming Loss is, the better classification performance is. Average Precision can be defined by the following:

Average Precision=

(15) $$\begin{array}{c} \frac{1}{\left|D\right|}\overset{\left|D\right|}{\underset{i=1}{\sum }}\frac{1}{\left|L_{i}\right|}{\sum^{}}_{l_{k}\epsilon L_{i}}\frac{\left\{l_{j}|rank\left(f_{i},l_{j}\right)\;\leq \;rank\left(f_{i},l_{k}\right),l_{j}\epsilon L_{i}\right\}}{rank\left(f_{i},l_{k}\right)}, \end{array}$$

where |*L*_*i*_| is the size of every label in the label set, and *rank*(f, l) records the rank of *l* after all labels are sorted in descending order. Average Precision reflects the average fraction of labels ranked higher than a specific label. Greater Average Precision indicates better classification performance. One Error can be defined as follows:

(16) $$\begin{array}{c} \text{One.Error}=\frac{1}{\left|D\right|}\overset{\left|D\right|}{\underset{i=1}{\sum }}\left\{\text{arg}\underset{l\epsilon L}{\text{max}}\left\{f\left(f_{i},l\right)\right\}\notin \;L_{i}\right\} \end{array}$$

One Error records the percentage of labels with the highest predicted value that are not contained by the relevant label set. The lower One Error is, the better classification performance is. Ranking Loss can be defined by the following:

Ranking Loss=

(17) $$\begin{array}{c} \frac{1}{\left|D\right|}\overset{\left|D\right|}{\underset{i=1}{\sum }}\frac{\left\{\left(l_{\text{j}},l_{\text{k}}\right)|f\left(f_{i},l_{\text{i}}\right)\leq \;f\left(f_{i},l_{\text{k}}\right),\;\left(l_{\text{j}},l_{\text{k}}\right)\epsilon L_{i}\times {\bar{L}}_{i}\right\}}{\left|L_{i}\|{\bar{L}}_{i}\right|}, \end{array}$$

where *f*(f, l) is the likelihood that *l* is the proper label of *f*, and ${\bar{L}}_{i}$ is the complementary set of *L*_*i*_. Ranking Loss reflects the average rank of these likelihoods. The lower Ranking Loss is, the better the classification performance is.

### 4.2. Datasets

The ten datasets are from Mulan Library [38], and Table 1 lists the detailed information of them. The domains of Corel5k, Flags, and Scene are images. Delicious, Medical, and Enron are text. GenBase is biology. The ten datasets contain various orders of magnitude, the number of features, and the number of labels. Additionally, datasets include different types of features, such as binary and polybasic. For experiments, every dataset has been divided into the training set and the test set by referring to the recommended size of the Mulan Library.

### 4.3. Analyze on Experiments

#### 4.3.1. Experiment 1

To prove the correctness of the chosen *α* and *β* in Section 3.4, we assign different values to *α* and *β* in CRMIL and test these values in all datasets. The Hamming Loss of results are then grouped by coefficients. The mean value of Hamming Loss in the same group is the standard value of the group. We choose the minimum value of the standard values as the normalizing number. Next, all standard values are divided by the normalizing number. Finally, we acquire the normalized results of all groups.

**Figure 3:**
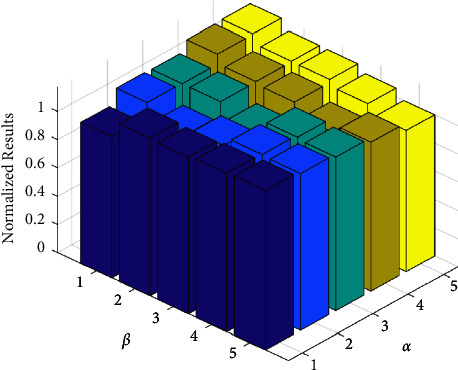
The standard results on different *α* and *β*.

The visualized results are represented in Figure 3. We can know that the corresponding bars are the lowest when *α* is equal to *β*. Moreover, if the ratio of *α* to *β* is larger, the results roughly become worse. This indicates CRMIL selects the best feature subset when *α* is equal to *β*. Therefore, the constant of formula (15) is suitable.

#### 4.3.2. Experiment 2

To explore the comparative performance of CRMIL on different datasets, we test CRMIL and the other eight multi-label feature selection algorithms on mentioned datasets. The results are evaluated by Hamming Loss, Average Precision, One Error, and Ranking Loss. Tables 2–5 demonstrate all experimental results in detail. These experimental results are obtained by averaging the results as they tend to stabilize after five simulations.

According to Hamming Loss, CRMIL performs better than the best-performing algorithms among the other eight algorithms on ten datasets. For example, CRMIL is 25.8%, 23.5%, and 12.8% better than AMI on Enron, Corel5k and Delicious, respectively. Compared with FSSL, CRMIL optimizes the target by 9% in Flags, 15.9% in Medical, and 9.9% in Scene. The average improvement on ten datasets is about 17.7%. In terms of the Average Precision, CRMIL improves the target by 0.0232 and 0.0204 on Flags and Scene, respectively. On Medical, GenBase, and Social, compared with the best-performance algorithm of the other eight algorithms (PMU, FSSL, and FSSL), CRMIL improves the results by 46.5%, 6.3%, and 4.3%, respectively. Although CRMIL slightly lower the result on Enron, the average result on ten datasets has been increased by around 10.2%. Taking One Error as the evaluation criterion, CRMIL reduces the percentage of errors by 32.6% and 6.2% on Flags and Scene, respectively. The average One Error on ten datasets has been increased by approximately 17.9%. For Ranking Loss, CRMIL performs well in all ten datasets, reducing the target by 83.3%, 44.8%, 12.1%, 9.8%, 7.4%, 6.9%, 5.0%, and 3.9% on Corel5k, Enron, GenBase, Flags, Yeast, Scene, Social, and Emotions, respectively, and the target becomes 0 on Delicious and Medical.

#### 4.3.3. Experiment 3

To study how many features should be selected when CRMIL can achieve stable experimental results, on Flags and Scene, we record the results with the increasing numbers of the selected features.

**Figure 4:**
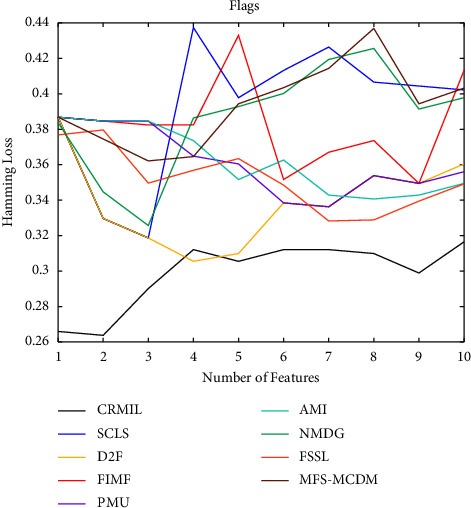
Hamming loss on flags.

**Figure 5:**
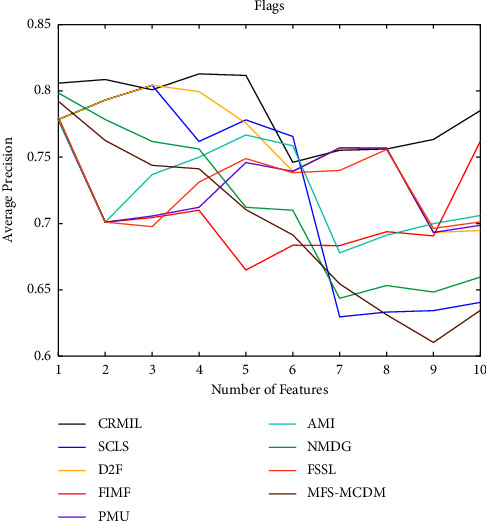
Average precision on flags.

**Figure 6:**
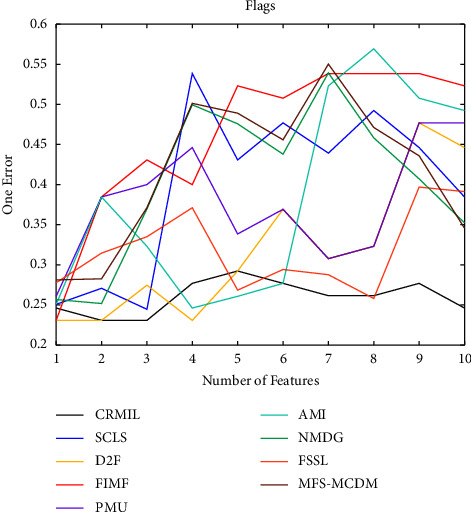
One Error on Flags.

**Figure 7:**
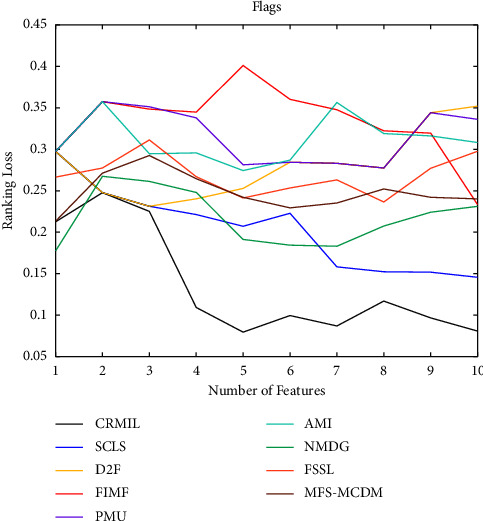
Ranking Loss on Flags.

**Figure 8:**
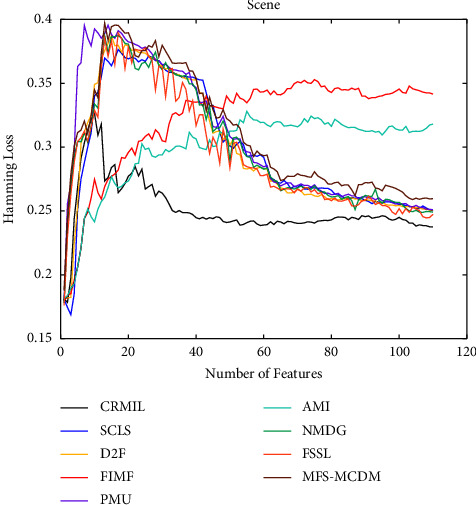
Hamming Loss on Scene.

**Figure 9:**
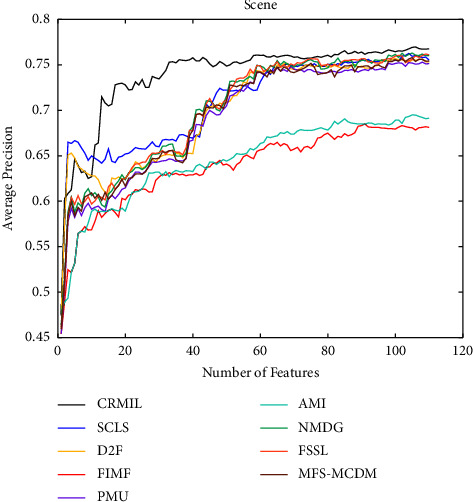
Average Precision on Scene.

**Figure 10:**
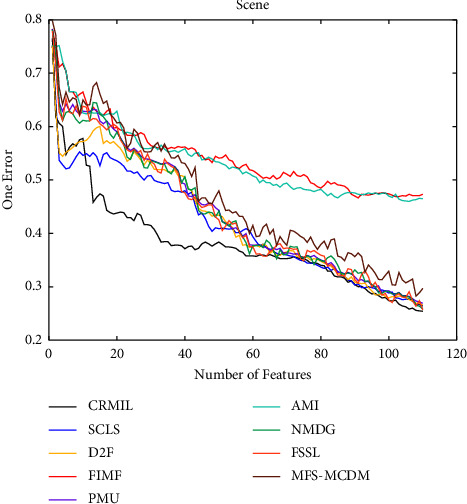
One Error on Scene.

**Figure 11:**
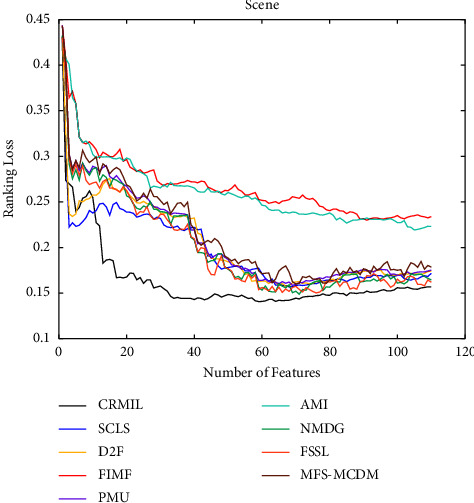
Ranking loss on scene.

In Figures 4–7 shows the experimental results of all the mentioned multi-label selection algorithms on Flags when different numbers of features are selected. Because there are 19 features in Flags, we choose the step of x-axis is 1 in Figures 4–7. The ranges of Hamming Loss, Average Precision, One Error, and Ranking Loss on Flags are (0.26, 0.44), (0.6, 0.85), (0.2, 0.6), and (0.05, 0.45), respectively. Similarly, Figures 8–11 details the experimental results on Scene. We select 20 is as the step of x-axis on Scene, because the maximum *k* is around 110 on this dataset. The ranges of Hamming Loss, Average Precision, One Error, and Ranking Loss on Scene are (0.15, 0.4), (0.45, 0.8), (0.2, 0.8), and (0.01, 0.45), respectively. On Flags and Scene, CRMIL has achieved good experimental results when the number of the selected features is 4 and 35, respectively. However, on Flags, SCLS, AMI, and FIMF cannot reach stable results when all features are selected, and the results of the other algorithms converge when the number of selected features is about 7. Furthermore, on Scene, most of the compared algorithms can get stable results if the number of selected features is around 60. This experiment indicates that CRMIL has a faster convergence. Compared with other algorithms, CRMIL can achieve better results and tend to be stable when the number of selected features is small.

#### 4.3.4. Experiment 4

To further explore the performance of CRMIL and investigate the improvement if algorithms consider the redundancy among labels, we make a comparative experiment regarding SCLS as the baseline. SCLS innovates multi-label feature selection by using mutual information without considering the redundancy among labels. If we can figure out the redundancy among labels and results improvement on every dataset, we can know the relation between label redundancy and results improvement by using CRMIL. To some extent, we can verify the efficiency of CRMIL on label-redundant datasets.

**Figure 12:**
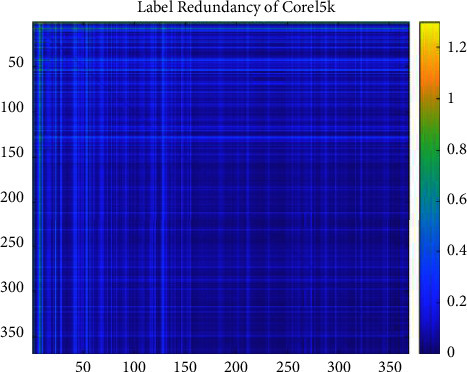
Label redundancy of Corel5k.

**Figure 13:**
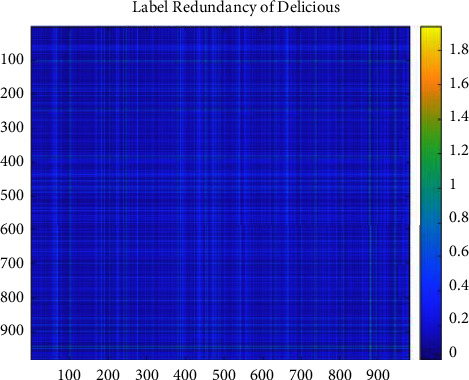
Label redundancy of Delicious.

**Figure 14:**
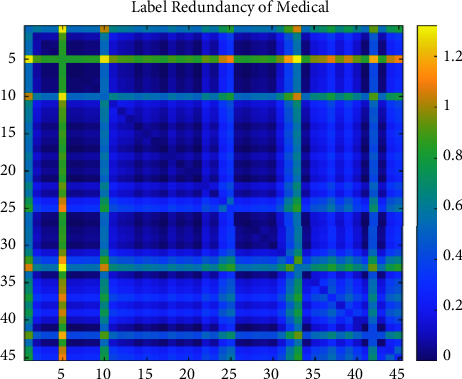
Label redundancy of Medical.

**Figure 15:**
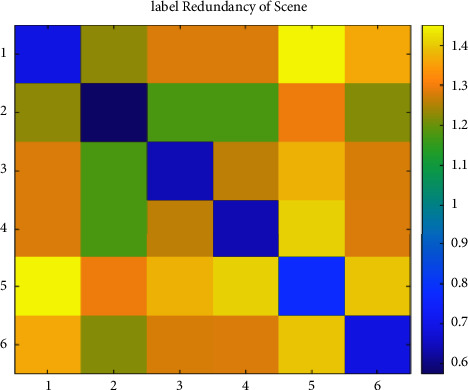
Label redundancy of Scene.

**Figure 16:**
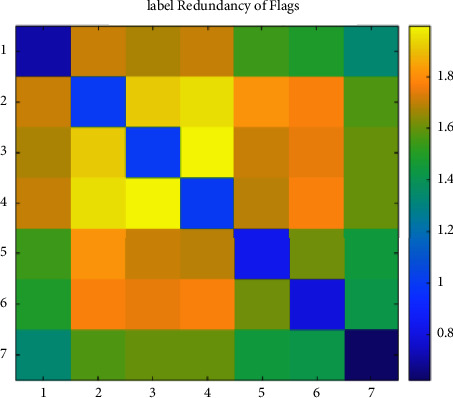
Label redundancy of Flags.

We set the mean of the optimization percentage of the experimental results of SCLS by CRMIL on Hamming Loss, Average Precision, One Error, and Ranking Loss as the results of improvement.  Table 6 details the mean value. Additionally, to understand the relation directly, we show both the redundancy between every two labels and the total label redundancy of every dataset. To illustrate the redundancy between every two labels, we use heatmaps (Figures 12–16) of five datasets. Both *x* and y axis represent labels in datasets and heat represents the redundancy between every two labels. The brighter color means the more redundancy among labels. From Figure 12–16, we can see that the heatmaps become brighter, which means the redundancy between every two labels of the five datasets increases in order of Corel5k, Delicious, Medical, Scene, and Flags. According to Table 6 and Figures 12–16, the proposed algorithm can get better results if more redundancy exists among labels. To describe the total label redundancy of datasets, we use formula (18) to represent the redundant value among labels.  Table 7 records the results.

(18) $$\begin{array}{c} \frac{1}{{\left|L\right|}^{2}}\underset{l_{i}\in L}{\sum }\underset{l_{j}\in L,l_{j}\neq l_{i}}{\sum }I\left(l_{i},l_{j}\right) \end{array}$$

**Figure 17:**
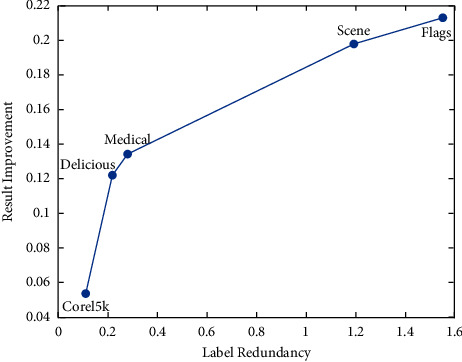
Relations between label redundancy and results improvement.

According to Table 6 and 7, the larger the redundancy among labels is, the better CRMIL will perform. As shown in Figure 17, the improvement of the results is roughly proportional to the redundancy among labels.

## 5. Conclusion and Future Work

In recent years, multi-label feature selection has become a hot topic. However, the existing multi-label feature selection algorithms have not fully considered the redundancy among labels. This paper proposes a new multi-label feature selection algorithm (CRMIL) that has considered the label set as the condition when computing the mutual information between features and labels.

To test the performance of this algorithm, we compare CRMIL with eight existing multi-label feature selection algorithms (SCLS, D2F, FIMF, PMU, AMI, NMDG, FSSL, and MFS-MCDM) on ten commonly used datasets (Corel5k, Delicious, Flags, Medical, Scene, Enron, GenBase, Social, Yeast, and Emotions) and use four evaluation criteria (Hamming Loss, Average Precision, One Error, and Ranking Loss) to evaluate results. Experimental results show that CRMIL performs better on various datasets, and the algorithm has a fast convergence speed. Furthermore, the greater the redundancy among labels is, the better the experimental results are.

However, according to the proposed multi-label feature selection algorithm, when the redundancy among labels is too dense, part of mutual information may not be counted in the final result, which can reduce the accuracy of the results. We may implement more high-dimension methods to partly overcome these challenges. In the future, we will take more special cases into account, study how to deal with the redundancy among labels more reasonably, and make the relevance between features and labels closer to the real value.

## Figures and Tables

**Algorithm 1:**
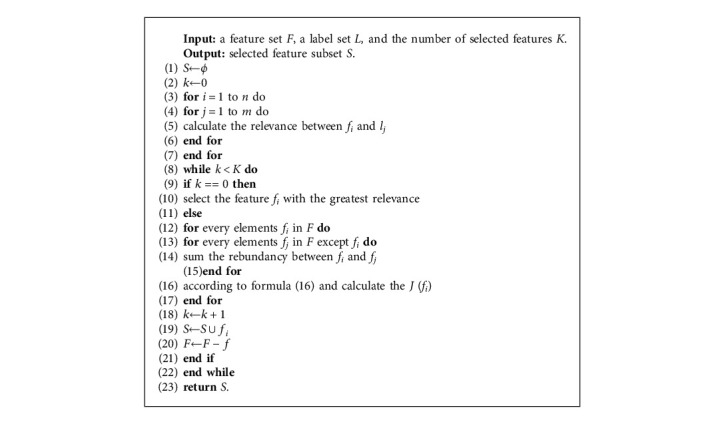
MCMI.

**Table 1:** Detailed information about datasets.

Dataset	Domain	^#^Instances	^#^Features	^#^Labels	^#^Training	^#^Test
Corel5k	Images	5000	499	374	4500	500
Delicious	Text	1075	500	983	862	213
Flags	Images	194	19	7	129	65
Medical	Text	978	1449	45	645	333
Scene	Images	2407	294	6	1211	1196
Enron	Text	851	1001	53	568	283
GenBase	Biology	662	1186	27	441	221
Social	Text	500	1047	39	333	167
Yeast	Biology	2417	103	14	1612	805
Emotions	Music	593	72	6	396	197

**Table 2:** Hamming Loss of results after applying multi-label feature selection algorithms on ten datasets.

Algorithms	CRMIL	SCLS	D2F	FIMF	PMU	AMI	NMDG	FSSL	MFS-MCDM
Corel5k	0.0104	0.0144	0.0235	0.0226	0.0227	0.0136	0.0141	0.0138	0.0194
Delicious	0.0170	0.0229	0.0336	0.0398	0.0374	0.0195	0.0217	0.0203	0.0231
Flags	0.3165	0.4022	0.3605	0.4132	0.3561	0.3496	0.3855	0.3520	0.3935
Medical	0.0211	0.0279	0.0386	0.0238	0.0319	0.0274	0.0253	0.0251	0.0283
Scene	0.2413	0.3009	0.3016	0.3363	0.3083	0.3126	0.2783	0.2679	0.2804
Enron	0.0723	0.0973	0.1027	0.0989	0.1031	0.0974	0.0847	0.0811	0.0873
GenBase	0.0052	0.0079	0.0062	0.0103	0.0091	0.0098	0.0074	0.0077	0.0101
Social	0.0424	0.0512	0.0563	0.0534	0.0712	0.0491	0.0472	0.0469	0.0507
Yeast	0.2319	0.2512	0.2579	0.2603	0.2591	0.2487	0.2449	0.2433	0.2496
Emotions	0.2613	0.2817	0.2833	0.3074	0.3096	0.2913	0.2804	0.2716	0.2775
Average	0.1219	0.1458	0.1464	0.1566	0.1651	0.1419	0.1390	0.1330	0.1420

**Table 3:** Average Precision of results after applying multi-label feature selection algorithms on ten datasets.

Algorithms	CRMIL	SCLS	D2F	FIMF	PMU	AMI	NMDG	FSSL	MFS-MCDM
Corel5k	0.0161	0.0139	0.0139	0.0138	0.0139	0.0139	0.0139	0.0140	0.0138
Delicious	0.0317	0.0238	0.0195	0.0203	0.0205	0.0292	0.0245	0.0258	0.0241
Flags	0.7852	0.6405	0.6947	0.7620	0.6986	0.7060	0.7122	0.7291	0.6972
Medical	0.1130	0.0553	0.0770	0.0553	0.0771	0.0589	0.0649	0.0692	0.0625
Scene	0.7487	0.7222	0.7085	0.6408	0.7048	0.6441	0.7256	0.7283	0.7204
Enron	0.5467	0.5138	0.5109	0.5017	0.5083	0.4892	0.5591	0.5337	0.5273
GenBase	0.8628	0.7549	0.7428	0.7025	0.7136	0.7813	0.8072	0.8114	0.7739
Social	0.5832	0.5479	0.5212	0.5153	0.5159	0.5427	0.5576	0.5593	0.5491
Yeast	0.7832	0.7582	0.7419	0.7404	0.7327	0.7493	0.7701	0.7679	0.7620
Emotions	0.7933	0.7701	0.7628	0.7634	0.7593	0.7631	0.7729	0.7814	0.7796
Average	0.5264	0.4801	0.4793	0.4716	0.4745	0.4778	0.5008	0.5020	0.4910

**Table 4:** One Error of results after applying multi-label feature selection algorithms on ten datasets.

Algorithms	CRMIL	SCLS	D2F	FIMF	PMU	AMI	NMDG	FSSL	MFS-MCDM
Corel5k	0.7021	0.7756	0.8884	0.8986	0.9083	0.7449	0.8325	0.7659	0.7835
Delicious	0.5821	0.5869	0.5869	0.6104	0.6104	0.6573	0.5819	0.5813	0.5839
Flags	0.2461	0.3847	0.4462	0.5231	0.4770	0.4924	0.4048	0.3195	0.4184
Medical	0.5057	1	0.5646	0.7058	0.5436	0.6288	0.5578	0.5351	0.5831
Scene	0.3837	0.4089	0.4340	0.5410	0.4432	0.5335	0.4176	0.4283	0.4478
Enron	0.3126	0.3892	0.3927	0.3914	0.4207	0.4126	0.3548	0.3420	0.4037
GenBase	0.2861	0.3318	0.3495	0.3512	0.3572	0.3436	0.3201	0.3158	0.4236
Social	0.2913	0.3147	0.3151	0.3261	0.3753	0.3428	0.3195	0.3017	0.3759
Yeast	0.3246	0.3401	0.3473	0.3572	0.3599	0.3487	0.3370	0.3318	0.3321
Emotions	0.3471	0.3722	0.3875	0.3812	0.3903	0.3689	0.3604	0.3557	0.3591
Average	0.3981	0.4904	0.4712	0.5086	0.4886	0.4874	0.4486	0.4277	0.4711

**Table 5:** Ranking Loss of results after applying multi-label feature selection algorithms on ten datasets.

Algorithms	CRMIL	SCLS	D2F	FIMF	PMU	AMI	NMDG	FSSL	MFS-MCDM
Corel5k	0.0001	0.0011	0.0082	0.0080	0.0079	0.0007	0.0008	0.0006	0.0010
Delicious	0	0.0026	0.0138	0.0155	0.0127	0.0049	0.0021	0.0019	0.0027
Flags	0.2156	0.2439	0.2477	0.3575	0.3682	0.3575	0.2176	0.2691	0.2483
Medical	0	0.0167	0.0140	0.0169	0.0139	0.3212	0.0148	0.0132	0.0162
Scene	0.1484	0.1781	0.1843	0.2625	0.1869	0.2603	0.1792	0.1561	0.1907
Enron	0.0812	0.1012	0.1218	0.1164	0.1359	0.1527	0.1194	0.0924	0.1274
GenBase	0.0327	0.0629	0.0726	0.0913	0.0897	0.0792	0.0613	0.0592	0.0623
Social	0.1329	0.1583	0.1672	0.1597	0.1591	0.1547	0.1517	0.1428	0.1581
Yeast	0.1923	0.2174	0.2278	0.2264	0.2401	0.2239	0.2104	0.2077	0.2065
Emotions	0.2383	0.2536	0.2752	0.2793	0.2811	0.2674	0.2548	0.2479	0.2507
Average	0.0764	0.0956	0.1037	0.12850	0.1218	0.1664	0.0934	0.0919	0.1008

**Table 6:** Results improvement based on SCLS.

Datasets	Corel5k	Delicious	Medical	Scene	Flags
Results improvement	0.053631321	0.121886728	0.133971278	0.197776749	0.213114755

**Table 7:** The results of label redundancy.

Datasets	Corel5k	Delicious	Medical	Scene	Flags
Label redundancy	0.112776503	0.216737498	0.279667978	1.19332472	1.553913238

## Data Availability

The data that support the findings of this study are available from the author upon reasonable request.
